# Administration of Low-Dose Dexmedetomidine Did Not Affect Acute Inflammatory Response after Cytoreductive Surgery Combined with Hyperthermic Intraperitoneal Chemotherapy: A Double-Blind Randomized Controlled Trial

**DOI:** 10.3390/jcm10143145

**Published:** 2021-07-16

**Authors:** Jiae Moon, Young Chul Yoo, Myoung Hwa Kim, Soyoung Jeon, Hye Ji Joo, Duk-Hee Chun, Na Young Kim

**Affiliations:** 1 Department of Anesthesiology and Pain Medicine, Anesthesia and Pain Research Institute, Yonsei University College of Medicine, 50-1 Yonsei-ro, Seodaemun-gu, Seoul 03722, Korea; ANSWLDO@yuhs.ac (J.M.); seaoyster@yuhs.ac (Y.C.Y.); HYEJIJOO@yuhs.ac (H.J.J.); 2 Department of Anesthesiology and Pain Medicine, Anesthesia and Pain Research Institute, Yonsei University College of Medicine, Gangnam Severance Hospital, 211 Eonju-ro, Gangnam-gu, Seoul 06273, Korea; KMH2050@yuhs.ac; 3 Biostatistics Collaboration Unit, Yonsei University College of Medicine, 50-1, Yonsei-ro, Seodaemun-gu, Seoul 03722, Korea; JSY0331@yuhs.ac; 4 Department of Anesthesiology and Pain Medicine, CHA Bundang Medical Center, CHA University School of Medicine, 59 Yatap-ro, Bundang-gu, Seongnam 13496, Korea

**Keywords:** cytoreductive surgery, hyperthermic intraperitoneal chemotherapy, dexmedetomidine, interleukin-6, inflammatory response

## Abstract

During cytoreductive surgery (CRS) with hyperthermic intraperitoneal chemotherapy (HIPEC), attenuation of inflammatory responses that increase susceptibility to postoperative complications, morbidity, and mortality is important. We aimed to evaluate whether intraoperative dexmedetomidine infusion impacted inflammatory response in patients undergoing CRS with HIPEC. Fifty-six patients scheduled for CRS with HIPEC were randomly assigned to the control (*n* = 28) and dexmedetomidine (*n* = 28) groups. The primary endpoint was the effect of dexmedetomidine on the interleukin-6 (IL-6) level measured at pre-operation (Pre-OP), before HIPEC initiation (Pre-HIPEC), immediately after HIPEC; after the end of the operation; and on postoperative day (POD) 1. In both groups, the IL-6 levels from Pre-HIPEC until POD 1 and the C-reactive protein (CRP) levels on PODs 1, 2, and 3 were significantly higher than the Pre-OP values (all Bonferroni corrected, *p* < 0.001). However, total differences in IL-6 and CRP levels, based on the mean area under the curve, were not detected between the two groups. The continuous intraoperative infusion of dexmedetomidine (0.4 μg/kg/h) in patients undergoing CRS with HIPEC did not significantly lower the inflammatory indices. Further dose investigative studies are needed to find the dexmedetomidine dose that provides anti-inflammatory and sympatholytic effects during HIPEC.

## 1. Introduction

Cytoreductive surgery (CRS) combined with hyperthermic intraperitoneal chemotherapy (HIPEC) has become an established therapeutic option for a growing number of malignancies including peritoneal carcinomatosis with different origins [1,2]. CRS with HIPEC eradicates all detectable tumor mass and combines it with a regional administration of high-dose chemotherapy to achieve a complete locoregional effect [1,2,3,4,5]. Given the long-lasting and complex surgical procedures, along with metabolic aberration and administration of local cytotoxic substances during the HIPEC phase, the risk of postoperative inflammatory response–induced complications is substantial; this may further affect postoperative morbidity and mortality even in patients without coexisting disease [4,6,7]. It has been reported that inflammatory response can play an important role not only in the development and progression of tumors but also in the response to treatment; hence, controlling inflammation may attenuate tumor promotion, dissemination, and recurrence [7,8].

Dexmedetomidine, a highly selective alpha-2 adrenergic receptor agonist with sedative and analgesic effect, has been used perioperatively. It not only reduces the demand for anesthetics and analgesics but also exhibits immunomodulating effects, such as suppression of sympathetic nerve hyperactivity and attenuation of stress response, as well as anti-inflammatory effects [9,10]. In recent years, the anti-inflammatory effect of dexmedetomidine has become more well-known—it has been shown to reduce the release of inflammatory factors and protect cellular immune function during the intraoperative period [11,12,13].

However, reports on the anti-inflammatory properties of dexmedetomidine in situations where excessive inflammatory reactions may occur are still insufficient [14,15,16]. HIPEC induces excessive inflammatory responses, and inflammation and immunosuppression may worsen owing to the combined use of HIPEC and surgical stimulation [7,17]. Thus, we hypothesized that intraoperative administration of dexmedetomidine could attenuate the inflammatory response in patients undergoing CRS with HIPEC.

## 2. Methods

### 2.1. Patients

This prospective, randomized controlled trial was approved by the Institutional Review Board and Hospital Research Ethics Committee of Severance Hospital, Yonsei University Health System, Seoul, Republic of Korea, in December 2017 (Protocol No. 4-2017-0372), and it was registered at http://clinicaltrials.gov (registration No. NCT03370588, accessed on 12 December 2017). Between December 2017 and May 2019, a total of 56 patients (aged 20–70 years) scheduled for CRS with HIPEC were enrolled after obtaining written informed consent. Patients were excluded if they met any of the following criteria: emergency operation; re-operation; combined surgery involving four departments; a history of cardiac diseases such as unstable angina, congestive heart failure, or valvular heart disease; ventricular conduction abnormality; prior pacemaker insertion; uncontrolled hypertension (diastolic blood pressure [BP] > 110 mmHg); bradycardia (heart rate [HR] < 40 beats/min); cerebrovascular disease (cerebral hemorrhage, cerebral ischemia); severe liver dysfunction (alanine aminotransferase >3 times the upper limit of normal); end-stage renal disease; use of an antiarrhythmic agent; or neurological or psychiatric impairment.

### 2.2. Study Design

Patients were randomly allocated to either the control group (*n* = 28) or dexmedetomidine group (*n* = 28) according to the computer-generated randomization code (19 June 2017). Patients in the dexmedetomidine group received a continuous infusion of dexmedetomidine (100 μg/mL in a 2-mL-vial; Hospira Worldwide, Seoul, Korea) at a rate of 0.4 µg/kg/h from anesthetic induction until the initiation of peritoneal closure [18]. The patients in the control group received the same volume of 0.9% normal saline as placebo. An anesthetist who did not participate in the data acquisition prepared the dexmedetomidine or saline. The group assignment was blinded to the surgeons, recovery nurses, attending anesthesiologists, patients, and the investigator.

### 2.3. Anesthetic Management

After arrival to the operating room, each patient underwent noninvasive BP measurement, electrocardiography, and oxygen saturation monitoring. Patient state index (PSI) was determined using a SedLine electroencephalograph sensor (Masimo Corp., Irvine, CA, USA). Following premedication with the intravenous (IV) bolus injection of 0.1 mg glycopyrrolate, anesthetic induction was initiated with 1.5–2 mg/kg propofol and 0.5 μg/kg remifentanil. After the loss of consciousness, tracheal intubation was facilitated with 0.6 mg/kg IV rocuronium. General anesthesia was maintained with 4–7% of desflurane with an adjuvant IV infusion of 0.05–0.2 µg/kg/min remifentanil by targeting a PSI within the range of 25–50. Mechanical ventilation was provided at a tidal volume of 7–8 mL/kg with 50% oxygen in air and a positive end-expiratory pressure of 5 cm H_2_O to maintain end-tidal carbon dioxide at 35–40 mmHg. Radial artery and central venous catheterization were performed in all patients, and hemodynamic parameters were collected via the Vigileo/FloTrac monitoring system (Edwards Lifesciences, Irvine, CA, USA). A continuous infusion of a vasopressor, phenylephrine or norepinephrine, was administered to maintain the mean arterial pressure (MAP) and HR within 20% of the basal preoperative value. Hydroxyethyl starch (6% 130/0.4) in a balanced electrolyte solution (Volulyte^®^, Fresenius Kabi AG, Bad Homburg, Germany) was used for perioperative fluid management accompanied by crystalloid infusion [19]. The body temperature was monitored continuously using an esophageal probe, and a Bair Hugger™ upper body airstream blanket (3M Deutschland GmbH, Neuss, Germany) with a forced-air warmer, hot IV line, and heated circuit was applied for warming during the CRS phase. During the HIPEC phase, active cooling was performed with a Bair Hugger™ upper body airstream blanket (3M Deutschland GmbH, Neuss, Germany) with a forced-air cooler, a cold IV line, and an unplugged heated circuit for the maintenance of normal body temperature.

### 2.4. Data Collection

The primary endpoint was the level of interleukin-6 (IL-6) measured at the following periods: pre-operation (Pre-OP), before the initiation of HIPEC (Pre-HIPEC), immediately after the end of HIPEC (HIPEC end), after the end of the operation (OP end), and on postoperative day (POD) 1. Blood samples were immediately centrifuged at 5000 rpm for 5 min at 4 °C, and the collected plasma was stored at −80 °C until the analysis. The plasma IL-6 levels were measured using the specific immunoassay kit (R&D, Cat. No. D6050, Minneapolis, MN, USA), and all tests were performed in duplicate. In addition, C-reactive protein (CRP), which is an inflammatory marker, was measured Pre-OP and on PODs 0, 1, 2, 3, and 30. Hemodynamic variables including MAP, HR, stroke volume variation (SVV), cardiac index (CI), and temperature were measured at Pre-OP, Pre-HIPEC, 1 h after HIPEC, HIPEC end, and OP end. Demographic and perioperative characteristics consisting of age, sex, body mass index, American Society of Anesthesiologists physical status, history of underlying diseases, cancer origin, peritoneal cancer index scores, completeness of cytoreduction, anesthesia time, operation time, intraoperative fluid balance, the total amount of norepinephrine infusion, and co-operation were assessed. Postoperative data included the number of patients who required ventilator care, length of hospital stay, and complications (pneumonia, acute kidney injury, ileus, obstruction, rebleeding, reoperation, and perforation).

### 2.5. Statistical Analysis

On the basis of a previous study, we calculated the required sample size [14]. To detect a 59.2 pg/mL difference in the IL-6 level between the dexmedetomidine and control groups, the power estimation analysis suggested that each group would need to include 24 patients, with 80% power at an alpha of 0.05. Finally, 28 patients were recruited in each group, thus allowing for a potential dropout rate of 15%.

Continuous variables were expressed as mean ± standard deviation, and categorical data were reported as the number of patients (percentage). For continuous and categorical variables, the independent *t*-test and chi-square test (or the Fisher’s exact test) were performed, respectively. Repeatedly measured data including IL-6, CRP, MAP, HR, SVV, and temperature were analyzed with a linear mixed model. If there were statistically significant differences, post hoc analysis with the Bonferroni correction was used to adjust for multiple comparisons. The mean area under the curve (AUC) was calculated for each group, and the difference in AUC between the groups was analyzed using an independent two-sample *t*-test. *p* values < 0.05 were considered statistically significant. Data analyses were performed using the SAS version 9.4 (SAS Inc., Cary, NC, USA).

## 3. Results

### 3.1. Patients

**Figure 1:**
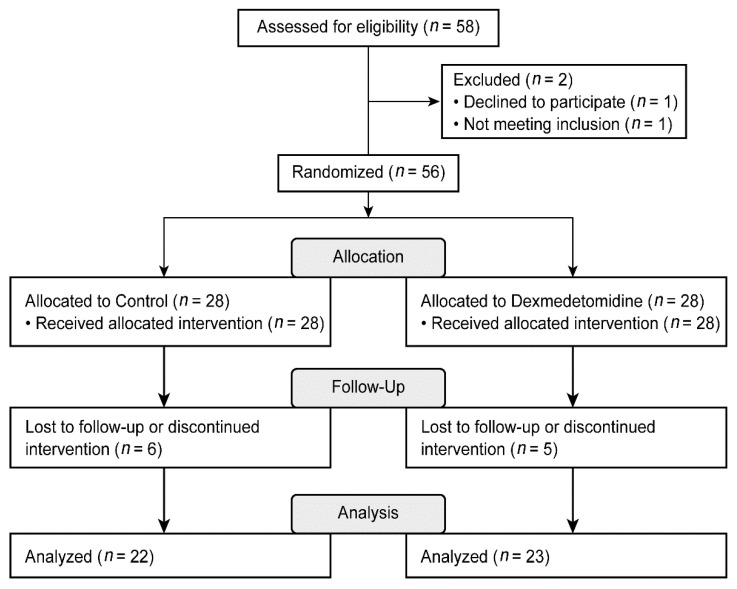
Consolidated Standards of Reporting Trials (CONSORT) flow diagram.

A total of 58 patients were assessed for eligibility, and one patient who declined to participate and another who did not meet the inclusion criteria were excluded. The remaining patients were randomly assigned to two groups. In each group, HIPEC was not performed in five patients. In the control group, one patient underwent laparoscopic CRS and was subsequently excluded from the study. Thus, a total of 45 patients were included in the final analysis (Figure 1).

### 3.2. Demographic and Perioperative Characteristics

The demographic and clinical characteristics of patients in both groups were similar and are presented in Table 1. As shown in Table 2, intraoperative parameters, including operation time, blood loss, and total requirements of norepinephrine, were not significantly different between the two groups. Five patients in each group required ventilator care after surgery. Postoperative complications including pneumonia, ileus, rebleeding, reoperation, and perforation were comparable between the two groups.

### 3.3. Inflammatory Indices: IL-6, CRP

**Figure 2:**
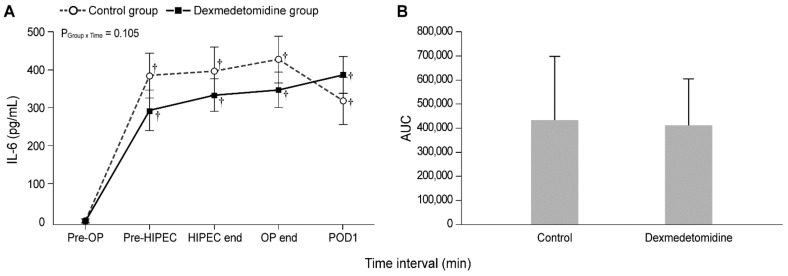
Perioperative actual changes in interleukin-6 (IL-6) levels (**A**) and the total differences in IL-6 levels via the analysis of AUC (**B**). Pre-OP, pre-operation; Pre-HIPEC, before the initiation of HIPEC; HIPEC end, immediately after the end of HIPEC; OP end, after the end of the operation; POD, postoperative day; AUC, area under the curve. Values are shown as mean ± standard deviation. † Bonferroni-corrected *p* < 0.001 versus Pre-OP in each group.

**Figure 3:**
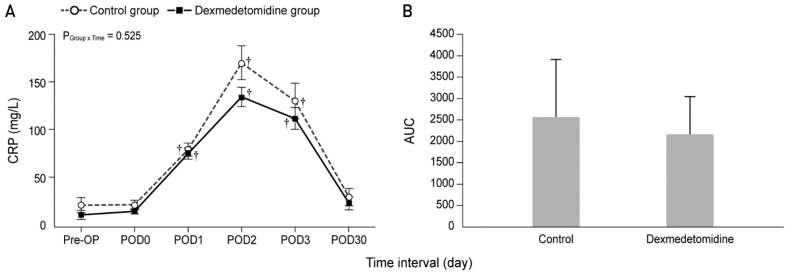
Perioperative actual changes in C-reactive protein (CRP) levels (**A**) and the total differences in CRP levels via analysis of AUC (**B**). Pre-OP, pre-operation; Pre-HIPEC, before the initiation of HIPEC; HIPEC end, immediately after the end of HIPEC; OP end, after the end of the operation; POD, postoperative day; AUC, area under the curve. Values are shown as mean ± standard deviation. † Bonferroni-corrected *p* < 0.001 versus Pre-OP in each group.

Figure 2 and Figure 3 show the actual changes (A) and total differences (B) in IL-6 and CRP levels. In both groups, the IL-6 levels from Pre-HIPEC until POD 1 and the CRP levels on PODs 1, 2, and 3 were significantly higher than the Pre-OP values (all Bonferroni corrected, *p* < 0.001). However, the IL-6 and CRP levels were not significantly different between the dexmedetomidine and control groups. In addition, total differences in the IL-6 and CRP levels between the two groups via the analysis of AUC showed no significant differences.

### 3.4. Hemodynamics: MAP, HR, SVV, CI, Temperature

**Figure 4:**
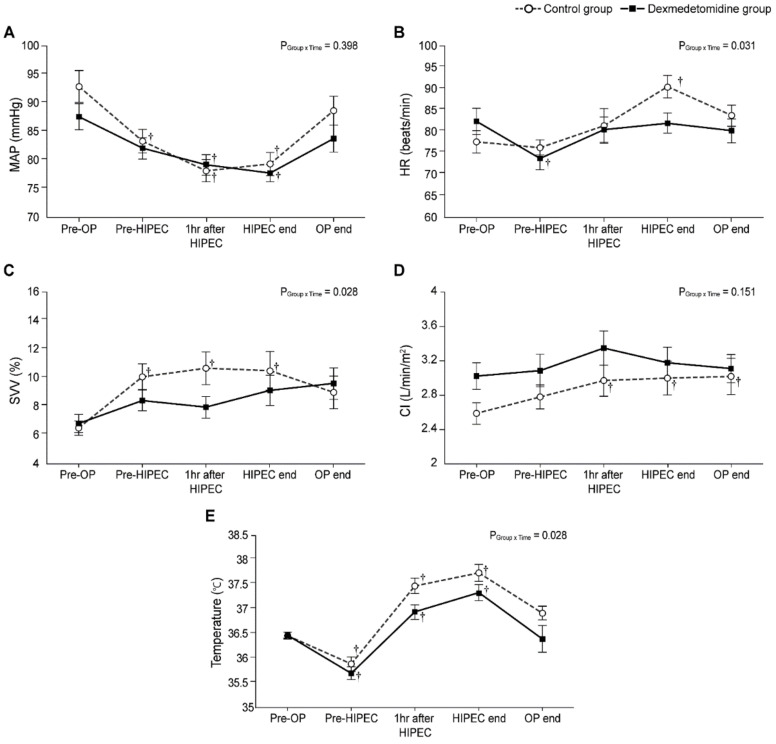
Intraoperative changes in (**A**) mean arterial pressure (MAP), (**B**) heart rate (HR), (**C**) stroke volume variation (SVV), (**D**) cardiac index (CI), and (**E**) temperature. Pre-OP, pre-operation; Pre-HIPEC, before the initiation of HIPEC; HIPEC end, immediately after the end of HIPEC; OP end, after the end of the operation; POD, postoperative day. Values are shown as mean ± standard deviation. † Bonferroni-corrected *p* < 0.05 versus Pre-OP in each group.

Figure 4 demonstrates the intraoperative changes in MAP, HR, SVV, CI, and temperature. The MAP at 1 h after HIPEC and at HIPEC end were significantly lower than the baseline values in both groups (Figure 4A). The linear mixed model analysis indicated a pattern of significant difference in HR, SVV, and temperature between the two groups (P_Group×Time_ = 0.031, 0.028, and 0.028, respectively). However, the post hoc analysis revealed that there was no statistical difference between the two groups at each time point. (Figure 4B,C,E). In the dexmedetomidine group, HR at Pre-HIPEC was significantly lower than the baseline value (Bonferroni corrected, *p* = 0.004); in the control group, HR at HIPEC end was significantly higher than the preoperative value (Bonferroni corrected *p* = 0.004). In addition, no patients experienced bradycardia (defined as HR < 40 beats/min). In the control group, the values of SVV at pre-HIPEC, 1 h after HIPEC, and HIPEC end were considerably higher than the baseline value (Bonferroni corrected *p* = 0.004, 0.003, and 0.020, respectively), and no statistically significant difference was seen in the dexmedetomidine group. No differences in CI were observed between the two groups. However, in the control group, the CI values 1 h after HIPEC, HIPEC end, and OP end were significantly higher than those prior to surgery (Bonferroni corrected *p* = 0.030, 0.018 and 0.017, respectively); no statistically significant differences were observed in the dexmedetomidine group (Figure 4D). In addition, temperatures in both groups showed a similar pattern; temperatures at Pre-HIPEC and during HIPEC were significantly lower and higher than the baseline value, respectively (all Bonferroni corrected *p* < 0.001).

## 4. Discussion

In this prospective, double-blind, randomized, controlled study, the effect of the continuous intraoperative administration of dexmedetomidine on inflammatory response in patients undergoing CRS with HIPEC was demonstrated. The administration of a clinical dose of dexmedetomidine (0.4 μg/kg/h) after induction of anesthesia until peritoneum closure did not significantly affect the inflammatory indices.

For patients with peritoneal carcinomatosis, CRS is designed to remove all visible macroscopic tumors within the abdominal and pelvic organs; this procedure can result in extensive tissue injuries, hypothermia, and fluid loss [3]. The HIPEC phase is aimed to eradicate residual microscopic tumor to prevent cancer recurrence via the regional administration of high-dose chemotherapy, which can induce hyperthermia and locoregional toxic damage to the abdominal organs [4,5]. These conflicting features of CRS and HIPEC (hypothermia vs. hyperthermia) can result in several complex physiological and biochemical changes, thus leading to multiple organ injuries [6]. The surgery itself induces a variety of stress responses and causes tissue damage, which activate the peripheral innate immune system, thus leading to cytokine cascade, the release of inflammatory mediators, and further aggravation of perioperative inflammation and immunosuppression [17]. Moreover, in HIPEC with CRS, cancer, surgery, hyperthermia, and high-dose chemotherapy are all strong stressors [17]. These stressors trigger endocrine, metabolic, hemodynamic, and inflammatory responses, especially during the operation and the early postoperative period [17,20], thereby increasing susceptibility to postoperative infection, delayed wound healing, multiple organ dysfunction, and morbidity and mortality [21]. Therefore, it is essential to implement strategies during CRS with HIPEC to modulate the balance of inflammatory responses, surgical stress, and immunity to potentially attenuate postoperative complications and improve postoperative clinical prognosis.

Dexmedetomidine provides anti-inflammatory and sympatholytic effects, which can be beneficial to patients perioperatively [9,10,11,12,13]. In addition, the organoprotective effects of dexmedetomidine against acute organ injury, such as injuries to the brain, lung, and kidney, have been well demonstrated in preclinical settings [22,23,24,25]. In recent years, the anti-inflammatory effect of dexmedetomidine has become more prominent—it is known to reduce the inflammatory response caused by endotoxin and inhibit the increase of tumor necrosis factor (TNF), IL-6, and neutrophils [26,27,28]. Among many cytokines, IL-6 is a major cytokine in the acute phase response that regulates cellular immunity by enhancing the innate immune system and protects against tissue damage [29]. The level of IL-6 is a marker for stress responses that reflects the degree of inflammation and tissue injury and is closely correlated with tumor occurrence and development [30,31]. IL-6 and TNF-alpha (TNF-α) concurrently stimulate the production of CRP, which is the most widely used inflammatory biomarker in clinical practice [32,33]. In the current study, IL-6 and CRP were evaluated as acute phase inflammatory markers, and it was revealed that serum IL-6 levels significantly and abruptly increased during the CRS with HIPEC; postoperative CRP levels were significantly increased until POD 3 compared with the baseline value in both groups. A human meta-analysis study by Wang et al. [12] reported a significant reduction in the levels of IL-6, TNF-α, and CRP and surgical stress response in patients who received perioperative dexmedetomidine. Dexmedetomidine suppressed the rapid increase of cytokine, TNF-α, and IL-6 levels in gastric cancer patients [34] and decreased IL-6 and CRP levels and the inhibition of T-lymphocyte subsets in colon cancer patients undergoing radical surgery [35]. Dexmedetomidine also reduced the upsurge of catecholamine release caused by stress or surgery [9]. These data indicate that dexmedetomidine can attenuate perioperative inflammation and protect the immune function of surgical patients. Therefore, dexmedetomidine was expected to attenuate metastatic progression and improve outcomes in cancer patients.

This study did not find any significant difference between the two groups in the inflammatory factors including IL-6 and CRP, even though other studies reported that dexmedetomidine significantly inhibited the secretion of inflammatory factors and the release of inflammatory mediators [12,13,36]. To minimize the adverse effects of dexmedetomidine, such as bradycardia and hypotension, a clinically approved low dose of dexmedetomidine was used in the current study; this dose seems to be insufficient to present the anti-inflammatory effect of dexmedetomidine in patients undergoing HIPEC given that the perioperative IL-6 levels in this study were two to sixfold higher than those seen in other non-HIPEC studies. HIPEC with CRS itself causes an immune response surge [7,17]. With the inflammation surge caused by HIPEC, the baseline inflammatory level was elevated to such an extent that it probably was not possible for the low-dose dexmedetomidine to suppress the inflammatory response level like in patients who did not receive HIPEC. Therefore, in cases where a massive inflammatory response is expected, such as during CRS with HIPEC, low-dose dexmedetomidine may not reduce the inflammatory response adequately.

Surgical stresses induce the release of catecholamines, which stimulate tumor growth [37]. Dexmedetomidine provides a sympatholytic effect on stress or surgery by suppressing the upsurge of catecholamines, and together with the anti-inflammatory effect, it is expected to reduce metastatic progression [38]. In the present study, dexmedetomidine significantly attenuated the increase in HR at the end of CRS with HIPEC, and any significant fluctuation was not shown in the dexmedetomidine group when compared with the Pre-OP value. By contrast, those in the control group showed significant fluctuations until the end of the CRS with HIPEC. However, dexmedetomidine can result in cardiovascular depression, including bradycardia or hypotension, and suppress the sympathetic tone in a dose-dependent manner. Atropine or ephedrine can be administered to treat these effects; however, in clinical situations such as hypovolemia or in patients with fixed stroke volume, the effect of dexmedetomidine can be deleterious [39,40]. Therefore, caution is required while using a high dose of dexmedetomidine.

This study had several limitations. First, all patients underwent CRS with HIPEC, but the diagnoses within the study population were heterogeneous. Second, the sample size was small, and the patients were followed up for a short period. Third, the pharmacological history of the patients was not evaluated; it could be a confounding factor. Additionally, the action of dexmedetomidine and vasopressors on the sympathetic axis could have influenced our results. Finally, instead of evaluating different doses of dexmedetomidine, only one infusion dose was evaluated in this study. Therefore, future studies that reflect these limitations are warranted.

In conclusion, the intraoperative infusion of 0.4 μg/kg/h dexmedetomidine in patients undergoing CRS with HIPEC did not significantly lower the inflammatory indices. Further dose investigative studies are necessary to find the dexmedetomidine dose that provides anti-inflammatory and sympatholytic effects at the time of HIPEC.

## Figures and Tables

**Table 1:** Demographic and preoperative characteristics.

Variables	Control Group (*n* = 22)	Dexmedetomidine Group (*n* = 23)	*p*-Value
Age, years	59.5 ± 13.0	53.4 ± 12.7	0.121
Female, *n*	18 (82%)	15 (65%)	0.208
Body mass index, kg/m^2^	23.6 ± 3.7	23.9 ± 3.1	0.779
ASA physical status			0.767
II	12 (52%)	13 (57%)	
III	10 (48%)	10 (43%)	
Hypertension	8 (36%)	5 (22%)	0.279
Diabetes mellitus	1 (5%)	1 (4%)	>0.999
Cancer origin			0.260
Colon origin	15 (68%)	19 (83%)	
OBGY origin	7 (32%)	4 (17%)	
PCI score	11.6 ± 5.6	12.6 ± 8.8	0.685
Complete cytoreduction	18 (82%)	22 (96%)	0.187

Notes: Data are presented as mean ± standard deviation or number of patients (proportion). Abbreviations: ASA, American Society of Anesthesiologists; PCI, Peritoneal Cancer Index; OBGY, Obstetrics and Gynecology.

**Table 2:** Perioperative profile.

Variables	Control Group (*n* = 22)	Dexmedetomidine Group (*n* = 23)	*p*-Value
Intraoperative variables			
Anesthesia time, min	636 ± 208	635 ± 208	0.984
Operation time, min	580 ± 205	581 ± 208	0.995
Intraoperative crystalloid, mL	5044 ± 2601	5920 ± 5345	0.487
Intraoperative colloid, mL	1089 ± 361	1152 ± 236	0.486
Transfused RBC, pack	2.0 ± 3.2	1.6 ± 2.1	0.594
Urine output, mL	1297 ± 879	1503 ± 849	0.429
Blood loss, mL	893 ± 778	873 ± 948	0.938
Total RI, unit	5.4 ± 7.1	4.9 ± 4.9	0.787
Intraoperative LEVO, µg/kg	22.1 ± 21.7	22.4 ± 20.3	0.962
Co-operation	16 (73%)	17 (74%)	0.928
Postoperative ventilator care	5 (23%)	5 (22%)	>0.999
Postoperative hospital stays, days	16 ± 11	16 ± 11	0.988
Follow up duration, days	573 ± 193	496 ± 209	0.207
Postoperative complication			
Pneumonia	1 (5%)	1 (4%)	>0.999
AKI	0 (0%)	0 (0%)	>0.999
Ileus	5 (23%)	6 (26%)	0.793
Obstruction	0 (0%)	0 (0%)	>0.999
Rebleeding	1 (5%)	0 (0%)	0.489
Reoperation	0 0%)	1 (4%)	>0.999
Perforation	0 0%)	1 (4%)	>0.999

Notes: Data are presented as mean ± standard deviation or number of patients (proportion). Abbreviations: RBC, red blood cells; AKI, acute kidney injury; RI, regular insulin; LEVO, norepinephrine.

## Data Availability

The datasets used and/or analyzed during the current study are available from the corresponding author on reasonable request.
